# The intercalated disc: a mechanosensing signalling node in cardiomyopathy

**DOI:** 10.1007/s12551-020-00737-x

**Published:** 2020-07-13

**Authors:** Mihai Pruna, Elisabeth Ehler

**Affiliations:** School of Cardiovascular Medicine and Sciences, King’s College London, BHF Research Excellence Centre, Randall Centre for Cell and Molecular Biophysics, Room 3.26A, New Hunt’s House, Guy’s Campus, London, SE1 1UL UK

**Keywords:** Cardiomyopathy, Mechanobiology, Cell-cell contact, Intercalated disc, Cytoskeleton

## Abstract

Cardiomyocytes, the cells generating contractile force in the heart, are connected to each other through a highly specialised structure, the intercalated disc (ID), which ensures force transmission and transduction between neighbouring cells and allows the myocardium to function in synchrony. In addition, cardiomyocytes possess an intrinsic ability to sense mechanical changes and to regulate their own contractile output accordingly. To achieve this, some of the components responsible for force transmission have evolved to sense changes in tension and to trigger a biochemical response that results in molecular and cellular changes in cardiomyocytes. This becomes of particular importance in cardiomyopathies, where the heart is exposed to increased mechanical load and needs to adapt to sustain its contractile function. In this review, we will discuss key mechanosensing elements present at the intercalated disc and provide an overview of the signalling molecules involved in mediating the responses to changes in mechanical force.

## Mechanical cues in the heart

Mechanical stimuli play a key role in both heart morphogenesis and in the mature heart. During heart development, mechanical forces orchestrate the molecular and cellular changes that transform the linear tubular heart into a multichambered machine with four valves (Lindsey et al. 2014). In the chick embryo, primordial heart contraction and the resulting pulsatile blood flow occurs before active oxygen transport is required (Burggren 2004), suggesting that contractile force is required not only for blood pumping but also for morphogenesis. Internal forces from cardiac contraction exert strain on the cell-cell junctions, whereas blood flow exerts both perpendicular (cyclic strain) and parallel forces (shear stress) to the vessel wall (Granados-Riveron and Brook 2012). In the developing heart, these mechanical forces are essential for shaping the chambered structure as well as for myofibrillogenesis (Geach et al. 2015), whereas in the fully formed heart, these cues are important in maintaining the structural and functional integrity of the myocardium. In cardiomyopathies, increased mechanical load triggers compensatory molecular and cellular changes temporarily allowing the myocardium to sustain pump function, but with time, these adaptive responses fail to meet the increased demand, resulting in cardiac dysfunction and heart failure (reviewed in Harvey and Leinwand 2011; McNally et al. 2013).

## Cardiomyocyte cytoarchitecture

**Fig. 1:**
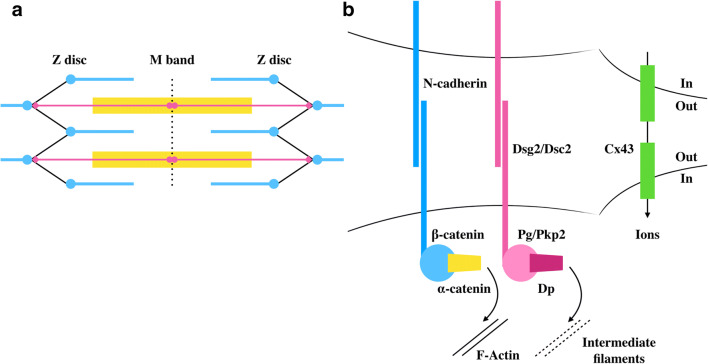
Simplified schematic representation of the two main cytoskeletal multiprotein complexes in cardiomyocytes **a** sarcomeres and **b** the intercalated disc. The sarcomere or the region between two Z-discs is the basic contractile unit of myofibrils, consisting of thin (actin and associated proteins, horizontal blue lines), thick (myosin and associated proteins, horizontal yellow block) and elastic (titin, horizontal pink line) filaments. Intercalated disc was originally described to consist of adherens junctions (blue and yellow symbols), desmosomes (pink symbols) and gap junctions (green symbols). The adherens junctions and desmosomes ensure transmission of force between neighbouring cardiomyocytes, allowing the heart to function in synchrony

Cells that make up the contractile tissue of the heart, the cardiomyocytes, are characterised by a highly regular architecture of cytoskeletal elements to ensure force generation and transduction with each heartbeat (reviewed in Ehler 2016). Cytoskeletal elements are organised into two major multiprotein complexes: the myofibrils and the intercalated disc (Fig. 1). Myofibrils, consisting of thin, thick and elastic filaments, contain the contractile machinery responsible for force generation. The basic unit of a myofibril is the sarcomere, defined as the region between two Z-discs (Fig. 1a). Thin filaments, composed of actin, tropomyosin and the troponin complex (troponin I, T, C), are anchored at the Z-disc predominantly via α-actinin (de Almeida Ribeiro et al. 2014). Thick filaments consist of myosin units, each containing two myosin heavy chains and two pairs of two myosin light chains (reviewed in Craig and Woodhead 2006). Myosin heads (or crossbridges) interact with actin, driving sarcomere contraction (Rayment et al. 1993). Myosin-binding protein C (MyBP-C) can associate with a subset of the myosin heads and regulate contraction (Kampourakis et al. 2014). Elastic filaments are made up of the giant protein titin, which stretches from the Z-disc to the M band (Fürst et al. 1988), a structure in the middle of the sarcomere defined molecularly by the presence of myomesin (reviewed in Lange et al. 2020). The myofibrils are anchored to the lateral plasma membrane at the Z-disc level through costameres (Samarel 2005) and at their ends by adherens junctions, a major component of the intercalated disc (reviewed in Bennett 2018). The intercalated disc (ID) is a highly specialised structure maintaining cell-cell adhesion and supporting transmission of contractile force and electrical signals from one cell to the next. It has been initially proposed that three distinct types of cell-cell contacts (Fig. 1b) can be distinguished at the intercalated disc: adherens junctions (fasciae adhaerentes) linking to actin filaments (i.e. myofibrils), desmosomes (maculae adhaerentes) anchoring intermediate filaments and gap junctions, ensuring electrical coupling (Forbes and Sperelakis 1985). More recently, a novel type of cell-cell contact, the area composita, which combines elements of both adherens junctions and desmosomes, has been defined for the adult mammalian heart (Franke et al. 2006). Several studies have contributed to a better understanding of how these elements intermingle. Plakoglobin (Pg) is a component of both adherens junctions and desmosomes (Witcher et al. 1996), while plakophilin2 (Pkp2) can interact with αT-catenin, an isoform expressed in the heart, brain and testis (Goossens et al. 2007; Li et al. 2012), suggesting a possible association between desmosomes and actin filaments. In addition, Pkp2 (Li et al. 2009; Oxford et al. 2007), desmocollin2 (Dsc2) (Gehmlich et al. 2011) and desmoglein2 (Dsg2) (Schinner et al. 2019) have been found to interact with the gap junction protein connexin43 (Cx43), creating a link between elements of the area composita and the electrical system.

## Mechanosensing and transduction at the ID

**Fig. 2:**
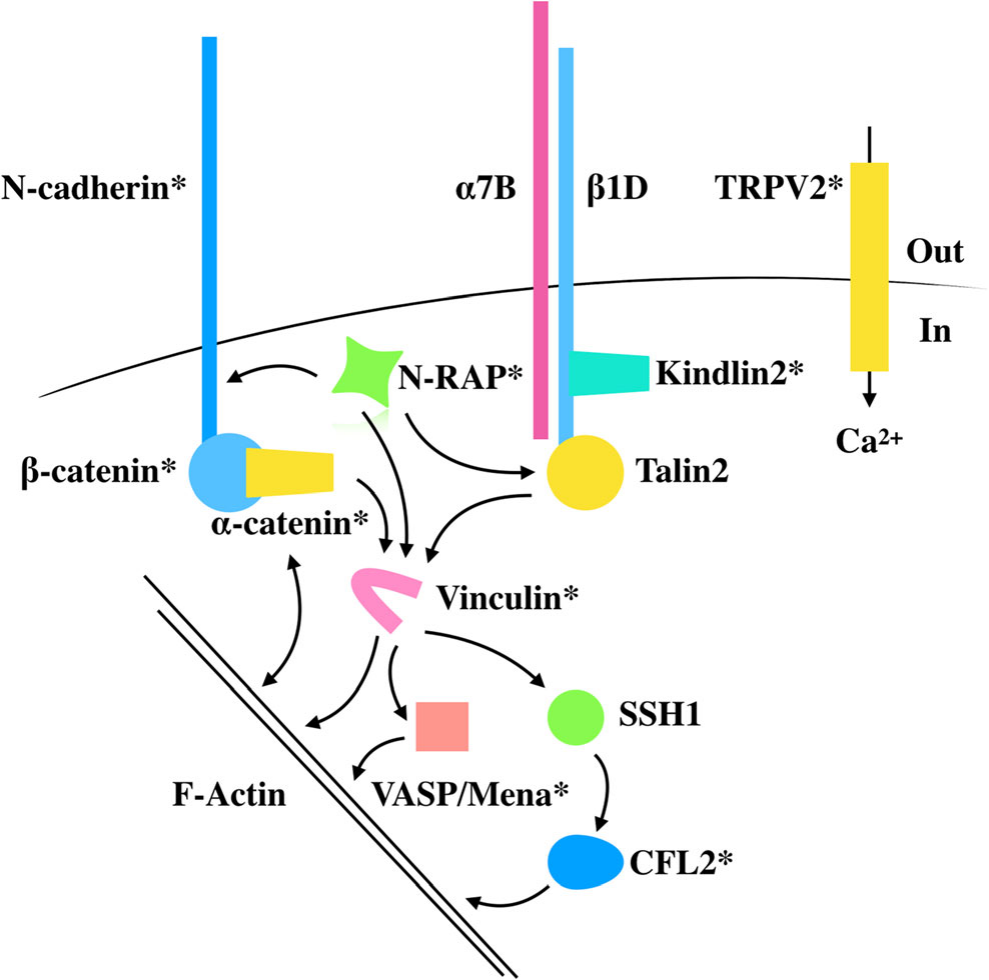
Simplified schematic representation of the protein complexes involved in force sensing and transduction at the intercalated disc. Molecules suggested to act as molecular switches in response to mechanical force are shown in yellow. Vinculin, in pink, is a key protein binding several factors involved in actin filament regulation. Vinculin phosphorylation on Tyr822 at adherens junctions was shown to be increased in epithelial MCF10a cells, but whether a similar modification is found in the heart remains to be elucidated. The TRPV2 channel is responsive to force and ensures the integrity of the intercalated disc. Proteins marked with * are affected in cardiomyopathies

Cytoskeletal elements are not only responsible for generating and transmitting contractile force, but several can also sense mechanical changes and transduce them into a biochemical signal, allowing the heart to regulate its output in response to internal or external mechanical stimuli (reviewed in Lyon et al. 2015)). In cardiomyocytes, established mechanosensors are found at the sarcomere and costamere (Lyon et al. 2015; Sit et al. 2019), but little is known about mechanosensing at the intercalated disc. Much of the understanding of intercalated disc force sensing and transduction (Fig. 2) has been interpolated from studies on the adherens junctions of epithelial cells (Ladoux et al. 2015; Merkel et al. 2019). Early embryonic cardiomyocytes, with cell-cell contacts around the entire surface and myofibrils parallel to the plasma membrane (Hirschy et al. 2006), resemble epithelial cells where actomyosin filaments form a circumferential belt in close vicinity to the cadherin-catenin complex (Ladoux et al. 2015). Similar to epithelial cell-cell contacts, mechanosensing and transduction at the intercalated disc can occur mostly through conformational changes in proteins of the adherens junctions complex: a change in the cytoplasmic domain of classical cadherins (Chopra et al. 2011) and exposure of the vinculin-binding domain (VBD) in α-catenin (Merkel et al. 2019). Whether these changes are independent or part of a signalling chain remains to be elucidated. Several components of integrin-based systems (Belkin et al. 1996, 1986; Dowling et al. 2008; Manso et al. 2013; Yi et al. 2003) are also located at the intercalated disc, but their precise role in mechanosensing at cell-cell contacts needs to be carefully investigated. More recently, changes in desmosomal proteins have also been described in response to increased mechanical force (Baddam et al. 2018; Price et al. 2018).

### Adherens junction components

#### N-cadherin

Classical cadherins (E-cadherin, N-cadherin) are large, modular, membrane spanning proteins of adherens junctions, located at ideal sites to sense and transduce changes in tension: on the extracellular side, they mediate cell-cell adhesion with another cadherin molecule on a neighbouring cell, whereas on the intracellular side, they are anchored to actin filaments via a complex of catenins (β, γ and α-catenin) (Maitre and Heisenberg 2013). Several groups showed that classical cadherins sense tension and can work as mechanoreceptors at cell-cell contacts (Borghi et al. 2012; Chopra et al. 2011). Borghi et al. constructed a hybrid FRET sensor, bearing a mechanosensitive domain flanked by Teal and Venus fluorescent proteins in the cytoplasmic domain of E-cadherin. Expressed in MDCK cells, the FRET efficiency of the sensor was significantly affected by actin polymerisation, actomyosin contractility or externally applied uniaxial stretch, thus providing direct evidence that tension from the cytoskeleton or external mechanical stimuli is sensed and transduced via the cytoplasmic domain of E-cadherin (Borghi et al. 2012). In cardiomyocytes, the only isoform expressed is N-cadherin (Volk and Geiger 1984). Conditional cardiac deletion of N-cadherin results in intercalated disc dissolution and myofibrillar disarray, associated with a loss of tension and impaired mechanical function (Kostetskii et al. 2005). To study the effect of cadherin-mediated stiffness sensing, a 2D system was employed where N-cadherin-Fc chimeras were cross-linked to polyacrylamide gels of varying stiffness onto which neonatal rat cardiomyocytes were cultured and several parameters such as spread area, myofibrillar alignment and cortical stiffness were measured (Chopra et al. 2011). Interestingly, by comparing cell-cell contact and cell-extracellular matrix (ECM) stiffness sensing, it was shown that cells grown on the N-cadherin substrate are more sensitive to lower forces (300 Pa) than those grown on ECM components, with a higher degree of cell spreading and myofibrillar alignment. Nevertheless, when substrate stiffness was increased to 5–10 kPa in cells grown on cadherin-coated substrate, myofibrils were randomly oriented and fragmented into distinct domains, in sharp contrast to cells grown on ECM-coated substrate where myofibrils were uniformly aligned across the myocyte. At even higher stresses (30 kPa), cells on both types of substrate were characterised by a more radial shape, myofibrillar disarray and increased cortical stiffness. Therefore, while both cell-cell and cell-ECM molecular machineries can sense mechanical force and induce changes in the morphology and internal organisation of cardiomyocytes, each may uniquely contribute to how cells adapt in various forms of cardiac myopathies (axial cell lengthening in dilated cardiomyopathy versus transverse cell lengthening in hypertrophic myocytes) (Chopra et al. 2011). It is important to mention that the role of classical cadherins as mechanoreceptors has been recently debated (Charras and Yap 2018). The transmembrane proteins are considered to only passively transmit contractile force between cells, yet more evidence is needed to clearly confirm the role of cadherins in mechanosensing and transduction.

#### α-Catenin

Cadherins bind through their cytoplasmic domain to β-catenin which links the cadherin molecules to the actin cytoskeleton via α-catenin (Buckley et al. 2014; Desai et al. 2013). α-Catenin has a modular structure, consisting of three main domains: an N-terminal domain-binding β-catenin, a modulatory domain (divided into MI, MII and MIII regions) binding vinculin via the vinculin-binding domain (VBD) in the MI region, but that also other interacts with other actin regulatory proteins, and a C-terminal domain-binding F-actin (Ishiyama et al. 2013; Kobielak and Fuchs 2004). α-Catenin has widely been accepted as a bona fide mechanoreceptor of adherens junctions, sensing and transducing force into a biochemical response via a conformational change in protein structure (Ishiyama et al. 2013; le Duc et al. 2010; Merkel et al. 2019; Rangarajan and Izard 2012; Seddiki et al. 2018; Thomas et al. 2013; Yao et al. 2014; Yonemura et al. 2010). A first insight into the role of α-catenin as a mechanosensor was provided in α-catenin-deficient R2/7 cells, which, despite expressing E-cadherin, lack various epithelial cell characteristics, including cell-cell junctions (Yonemura et al. 2010). Interestingly, expression of α-catenin not only completely restored cell-cell contacts but also recruited vinculin to the plasma membrane. Decreasing force by inhibiting actomyosin contractility resulted in the disappearance of the vinculin, but not the α-catenin signal from the junctions. To characterise the force-dependent mechanism behind this interaction, several α-catenin truncations were created and expressed in R2/7 cells. Expression of an α-catenin mutant lacking the F-actin binding C-terminal domain failed to recruit vinculin at the plasma membrane. Nevertheless, if α-catenin was further truncated to consist of only the N-terminal domain and the MI region and expressed in cells, vinculin did accumulate at the plasma membrane irrespective of a decrease in force. These experiments led to two key assumptions: (1) the MII and MIII regions keep α-catenin in a closed, autoinhibited state unable to bind vinculin, and (2) the C-terminal domain is a key part of the molecule involved in force sensing, inducing conformational changes in the molecule that release the autoinhibition, rendering α-catenin in an open state and able to bind vinculin. These assumptions were validated by the use of an antibody (α18) against a region of α-catenin located between the VBD and the inhibitory regions. In several endogenously expressing α-catenin epithelial cells, the α18 antibody stained adherens junctions, colocalised with vinculin and was in close proximity to myosin II. Decreasing mechanical force by inhibiting actomyosin contractility, disrupting actin filaments or disrupting cadherin-mediated adhesion resulted in the disappearance of both α18 and vinculin staining, suggesting that mechanical force is required to unmask the VBD and the α18 epitope (Yonemura et al. 2010). An increased recruitment of α-catenin, vinculin and F-actin at cell-cell contacts and an increased signal of the α18 antibody were also observed when MDCK cells were plated on stiff substrates (35 kPa) (Seddiki et al. 2018).

How tension changes the conformation of the protein has been later described by solving the crystal structure of autoinhibited α-catenin at 6.5-Å resolution, together with the determination of the crystal structure of the VBD bound to the corresponding domain of vinculin at 2.7-Å resolution (Ishiyama et al. 2013; Rangarajan and Izard 2012). In the autoinhibited state, the helical bundles of MI, MII and MIII are organised into a λ-shaped conformation, with the MIII region preventing access to the VBD in MI (Ishiyama et al. 2013). Upon tension sensing by the C-terminal domain binding to F-actin, the MI region ‘unfurls’, allowing vinculin to bind the VBD (Rangarajan and Izard 2012). In the heart, conditional deletion of αE-catenin, the major isoform, resulted in large, highly convoluted intercalated discs, disorganised myofibrils and a propensity to ventricular rupture after myocardial infarction (Sheikh et al. 2006). Interestingly, in these mice, the expression levels of vinculin were reduced with a complete loss of vinculin at the intercalated disc, but not at the costameres, suggesting the importance of the α-catenin-vinculin interaction at adherens junctions in maintaining structural and functional integrity of the heart. In the muscle LIM protein (MLP, also called Csrp3)-knockout mouse, a model for dilated cardiomyopathy, the levels of α-catenin, vinculin and F-actin were increased at the intercalated disc, which could well be an adaptive response in supporting the increased mechanical load of the failing heart (Ehler et al. 2001).

#### Vinculin

Vinculin is an actin binding and regulatory protein, being recruited to both cell-cell and cell-matrix multiprotein complexes involved in mechanotransduction (Bays and DeMali 2017; Carisey and Ballestrem 2011; Huveneers and de Rooij 2013). In cardiomyocytes, vinculin and its cardiac and smooth muscle-specific isoform, metavinculin, localise at both the intercalated disc and costameres (Belkin et al. 1988). Cardiac-specific vinculin knockout mice developed left ventricular dysfunction that evolved into dilated cardiomyopathy (Zemljic-Harpf et al. 2007). At the ultrastructural level, the intercalated discs of these vinculin-deficient hearts appeared highly serrated, with a low electron density and a significant separation from myofibrils (Zemljic-Harpf et al. 2007). Several mutations in metavinculin had been associated with dilated cardiomyopathy, with the Arg975Trp mutation resulting in large aggregates of actin filaments together with irregular and fragmented intercalated discs, supporting the notion that disrupted impaired contractile force transmission can lead to dilated cardiomyopathy (Olson et al. 2002). In isolated neonatal mouse cardiomyocytes, inhibition of actomyosin contractility resulted in a significant reduction of the vinculin signal at the intercalated disc, confirming the model previously described in epithelial cells where force is required to induce a conformational change in α-catenin to allow recruitment of vinculin at the junctions (Merkel et al. 2019).

However, which other factors trigger vinculin-specific localisation at the cell-cell contacts and what is the role of vinculin once recruited to the adherens junctions complex? Phosphorylation of vinculin on Tyr822 in cell-cell, but not cell-matrix adhesions, was elevated when force was applied to epithelial MCF10a cells, suggesting that post-translational modification plays a role in how vinculin differentially supports mechanotransduction at cell-cell and cell-matrix adhesions (Bays et al. 2014). Once recruited to the adherens junctions, it has been proposed that vinculin can stiffen the cadherin-catenin complex (le Duc et al. 2010; Seddiki et al. 2018; Yonemura et al. 2010). Indeed, fluorescence recovery after photobleaching (FRAP) experiments in R2/7 cells expressing an orange fluorescent protein (OFP)–tagged α-catenin which induced the formation of cell-cell contacts and recruited vinculin to the junctions revealed that the mobility of α-catenin is increased by actomyosin contractility inhibition (Yonemura et al. 2010). Importantly, the dynamics of the protein were restricted by expression of an OFP-tagged α-catenin construct constitutively binding vinculin, suggesting that tension-dependent vinculin recruitment stabilises α-catenin at the cell-cell contacts and may contribute to the reinforcement in F-actin binding. Closer examination of vinculin recruited at cell-cell contacts reveals that it does not uniformly colocalise with E-cadherin all over the junctions but is restricted to discrete sites in which the cadherin-catenin complexes contact F-actin bundles (le Duc et al. 2010). Interestingly, knockdown of vinculin reduced the levels of phosphorylated myosin light chain (pMLC) (le Duc et al. 2010), as well as steady-state F-actin and barbed-end content from sites of cell-cell contact (Leerberg et al. 2014) supporting the notion that vinculin is important for cytoskeleton remodelling at the junctions (le Duc et al. 2010). In neonatal mouse cardiomyocytes lacking endogenous N-cadherin, several green fluorescent protein (GFP)–tagged N-cadherin hybrids linked to various domains of α-catenin that differed in F-actin binding and vinculin recruitment were expressed to rescue the lack of N-cadherin, and the structure of myofibrils and intercalated discs was analysed by thin section transmission electron microscopy (Merkel et al. 2019). Importantly, the intercalated disc and myofibrillar arrangement were rescued in cells in which an N-cadherin hybrid was connected to the MI domain of α-catenin (thus allowing constitutive vinculin binding), together with either the MII region or the F-actin binding domain of α-catenin, but not in cells expressing the F-actin domain and the MII region of α-catenin (in which the molecule is unable to recruit vinculin to cell-cell contacts), suggesting that vinculin presence at the intercalated disc is required for connecting adherens junctions to contractile actin (Merkel et al. 2019).

An intriguing interplay is established where myosin II (and thus tension generation) is required for vinculin recruitment at cell-cell junctions, and once recruited, vinculin can stabilise the actomyosin contractile machinery. A paradox of this feedback system is the ability of myosin II to sever F-actin filaments, thus restricting actin assembly (Medeiros et al. 2006; Wu et al. 2014). One possible way to regulate this effect is to consider the ability of vinculin to act as a scaffold to recruit actin regulatory proteins such as Mena and WASP to promote actin assembly at the cell-cell contacts (Leerberg et al. 2014). Mena and WASP are suggested to regulate the actin cytoskeleton by acting as anti-capping proteins, allowing actin filament elongation (Bear and Gertler 2009). In cardiomyocytes, Mena is not only found at the intercalated disc (Aguilar et al. 2011), but its expression is unregulated in heart failure (Blaxall et al. 2003), suggesting that it may play a similar role in the heart. Nevertheless, despite being located at the cell-cell contacts, no significant change in Mena recruitment to the intercalated disc was found when the hybrid N-cadherin-GFP-MI-MII, which constitutively binds vinculin, was expressed in neonatal mouse cardiomyocytes lacking endogenous N-cadherin, compared with constructs unable to bind vinculin, suggesting that while Mena may play a role in actin assembly, its recruitment is limited and does not correlate with vinculin levels (Merkel et al. 2019). However, VASP, which is also found at the intercalated disc, might compensate the function of Mena in cardiomyocytes (Eigenthaler et al. 2003). Cardiac-specific overexpression of a dominant negative VASP which displaced endogenous VASP and Mena from the cell-cell contacts resulted in mice with disorganised intercalated discs and disrupted adherens junctions, accompanied by dilated cardiomyopathy, supporting a role for VASP in maintaining structural and functional integrity of the cell-cell contacts (Eigenthaler et al. 2003).

A proteomic screen in non-contracting and contracting rat cardiomyocytes identified the slingshot protein phosphatase (SSH1) as a vinculin-binding partner in contracting cells (Fukuda et al. 2019). Indeed, the vinculin-SSH1 interaction was increased by cyclical stretch and abolished when cardiac contractility was inhibited, suggesting that the interaction is force-dependent (Fukuda et al. 2019). SSH1 can dephosphorylate and activate the actin regulator cofilin (CFL), which severs F-actin filaments to supply monomeric G-actin and promote filament reassembly (Ohashi 2015). Importantly, cyclic stretch decreased CFL phosphorylation, whereas inhibition of cardiac contractility, vinculin depletion or expression of a dominant negative form of SSH1 results in increased phosphorylation and inactivation of CFL, suggesting that the vinculin-SSH1 axis can trigger CFL activation in cardiomyocytes exposed to increased mechanical forces. Interestingly, in idiopathic DCM, fibrillar plaques have been found in almost three-quarters of patients (Gianni et al. 2010). The precursors of these plaques, termed pre-amyloid oligomers (PAO), were shown to contain CFL2, myosin light chain II and α-cardiac actin (Subramanian et al. 2015). The levels and phosphorylation of CFL2 in idiopathic DCM patients were increased, indicating that in these patients, CFL activity is reduced both by phosphorylation and sequestration within the PAO (Subramanian et al. 2015). Interestingly, neonatal rat cardiomyocytes infected with a phosphomimetic CFL2 adenovirus displayed ‘stress-like’ fibre structures and reduced contractility compared with cells infected with wild-type adenovirus, suggesting that excess actin filament formation is detrimental for contraction (Subramanian et al. 2015). It may be that CFL recruitment to the vinculin-SSH1 complex is a protective mechanism in response to increased mechanical load since decreased CFL activity is associated with DCM (Fukuda et al. 2019). It will be interesting to study the precise localisation of CFL in cardiomyocytes, but immunofluorescence microscopy of human hearts revealed that CFL2 is present predominantly at the intercalated disc (Subramanian et al. 2015), further supporting its role in mechanotransduction at this specialised site.

The plasma membrane at cell-cell contacts can itself act as a force-sensing element (Charras and Yap 2018; Dorland and Huveneers 2017; Huveneers and de Rooij 2013). In endothelial cells, several agonists can induce the formation of vinculin-enriched focal adhesions junctions (FAJs), which are transversally oriented to the cell-cell plane, and are distinct from linear adherens junctions (Huveneers and de Rooij 2013). The formation of FAJs is prevented when actomyosin contractility is inhibited, suggesting that hormone-induced membrane remodelling is dependent on cytoskeletal forces (Huveneers and de Rooij 2013). Since FAJs appear as fingerlike projections with increased curvature of the membrane, this could allow BAR domain proteins to be recruited to the cell-cell junctions (Charras and Yap 2018). In endothelial cells, the BAR domain containing protein pacsin2 was found at the trail of FAJs where it was shown to prevent VE-cadherin internalisation (Dorland and Huveneers 2017). Whether zigzag adhesions can form in cardiomyocytes in response to mechanical force remains to be investigated, but it is well known that the plasma membrane at the intercalated disc appears more convoluted in the hearts of old monkeys (Forbes and Sperelakis 1985) and mouse models of dilated cardiomyopathy (Ehler et al. 2001), indicating that a similar mechanism might be involved (Perriard et al. 2003).

#### N-RAP

Another protein with a possible role in mechanosensing and transduction at the intercalated disc is nebulin-related-anchoring protein (N-RAP), a striated muscle-specific protein which was proposed to be part of the mechanical link between the intercalated discs and myofibrils (Lyon et al. 2015; Zhang et al. 2001). Via its N-terminal LIM domain, N-RAP can bind talin and α-actinin, while via its C-terminal domain, it was shown to bind vinculin and actin (Luo et al. 1999), as well as MLP (Ehler et al. 2001). Ultrastructural analysis of adult mouse hearts reveals that whereas vinculin is present at the membrane of adherens junctions, N-RAP is found near the membrane, close to terminal actin filaments that link the myofibrils to the plasma membrane (Zhang et al. 2001). Fractionation of mouse hearts revealed that N-RAP co-purified with key constituents of the intercalated disc (N-cadherin, β1D-integrin, vinculin, talin) and myofibrillar proteins (α-actinin and myosin heavy chain). Importantly, detergent extraction and sucrose gradient separation which removed residual myosin and actin, but also vinculin and β1D-integrin, left a fraction enriched in N-RAP together with N-cadherin, talin and α-actinin, suggesting that N-RAP is connected to the junctions predominantly via the cadherin system. Nevertheless, since talin can also bind β1D-integrin (see below), N-RAP might play a role in cross-linking the integrin and cadherin systems at the intercalated disc (Zhang et al. 2001). In the hearts of MLP knockout mice, an early upregulation of N-RAP expression was found, suggesting that increased N-RAP levels could be one of the earliest hallmarks of dilated cardiomyopathy (Ehler et al. 2001). Overexpression of NRAP in the murine heart results in right ventricular cardiomyopathy, with little effect on the left ventricle and intercalated disc structure, questioning the role of N-RAP in the disease (Lu et al. 2011). A rare truncating mutation in N-RAP (Arg1502*) which severely reduces protein levels was found in a patient who developed dilated cardiomyopathy in response to a viral illness (Truszkowska et al. 2017). The same homozygous mutation was carried by his healthy sibling, suggesting that the Arg1502* N-RAP mutation has low penetrance and might require other factors to cause disease (Truszkowska et al. 2017).

### Desmosome components

The desmosome is a multiprotein complex composed of a membrane spanning desmosomal cadherin (desmoglein or desmocollin) whose extracellular domain interacts with another desmosomal cadherin molecule on a neighbouring cell, while the intracellular domain links to intermediate filaments via the cytosolic armadillo proteins Pg (γ-catenin) and Pkp and the plaque protein desmoplakin (DP) (Fig. 1b, reviewed in Green et al. 2019). Desmosomes provide mechanical strength in organs exposed to increased shear stress, such as the skin and the heart (Bolling and Jonkman 2009). While in the skin, multiple desmosomal cadherins and plakophilins are expressed, in the heart, isoforms are restricted to Dsg2, Dsc2 and Pkp2, along with Pg and DP (Patel and Green 2014). Mutations in desmosomal proteins are found in nearly half of patients with arrhythmogenic cardiomyopathy (AC) (Asimaki et al. 2007; Basso et al. 2012; Gerull et al. 2004; Klauke et al. 2010; Pilichou et al. 2006; Rampazzo et al. 2002; Syrris et al. 2006), a disease characterised by progressive replacement of the ventricular myocardium with adipose and fibrous tissue, resulting in ventricular arrhythmias and sudden cardiac death (Vimalanathan et al. 2018). Interestingly, mutations in the area composita protein αT-catenin are also associated with AC (van Hengel et al. 2013). At the ultrastructural level, the intercalated disc of patients with the disease showed shorter desmosome-like structures, which are present at abnormal locations and display a much wider desmosomal gap (Basso et al. 2006).

Recent studies have shown that desmosomes are not rigid but can act as mechanosensory structures (Baddam et al. 2018; Price et al. 2018). Expression of Dsg2 bearing a C-terminal tension–sensitive FRET sensor in human-induced pluripotent stem cell (hiPSC)–derived cardiomyocytes revealed a significant change in the FRET signal during cellular contraction (Baddam et al. 2018), suggesting that Dsg2 can sense the internal contractile forces generated with each heartbeat. By contrast, expression of a DP FRET-based tension sensor in epithelial cells experienced significant mechanical load only when cells were exposed to external forces and little or no load from contractile forces generated by individual cells (Price et al. 2018). A possible explanation for these contrasting observations is an alternative connection between desmosomal cadherins and the actin cytoskeleton, possibly mediated by Pkp2/αT-catenin in the heart (Goossens et al. 2007). It will be interesting to determine whether DP and other desmosomal components can sense mechanical load during cardiomyocyte contraction and, in particular, whether αT-catenin can function as a mechanosensor while interacting with Pkp2.

### Integrin components at the ID

Integrins are large, heterodimeric transmembrane proteins involved in mechanotransduction at the ECM-cell contacts. In cardiomyocytes, they are mostly found at the costameres (Ross and Borg 2001). Interestingly, in the adult mouse cardiac muscle, β1D-integrin, the major isoform expressed, was found to be localised not only at the costameres but also at the intercalated disc, with α7B integrin being the major binding partner (Belkin et al. 1996). Phenylephrine stimulation of neonatal rat ventricular myocytes results in a significant increase in β1D-integrin expression, accompanied by an increase in atrial natriuretic peptide (ANP), a marker of cardiac hypertrophy (Pham et al. 2000). A change in subcellular localisation of β1D-integrin was also observed, from diffuse cytosolic punctae to an increase signal at the Z-disc. One flaw of this study is that plated cells were too scarce to allow formation of cell-cell contacts, thus not allowing the study of the protein at the intercalated disc in response to α1-adrenergic stimulation. Nevertheless, the authors observed an increased phosphorylation of focal adhesion kinase (FAK), a non-receptor tyrosine kinase, in response to phenylephrine treatment, and coimmunoprecipitated β1D-integrin and FAK from cardiac myocyte protein extracts, suggesting that FAK could be a downstream effector of integrin signalling (Pham et al. 2000). Indeed, in spontaneously hypertensive heart failure (SHHF) rats, FAK was found to translocate to the intercalated disc where its autophosphorylation on Tyr397 was increased (Yi et al. 2003). In mice, mechanical stress triggered by pressure overload resulted in cardiac hypertrophy and increased levels of FAK phosphorylation, together with increased levels of β1D-integrin and its binding partner, α7B integrin, particularly at the intercalated disc, reflecting the adaptation of the heart to increased mechanical load (Babbitt et al. 2002). Nevertheless, studies with human samples have revealed that the protein levels of β1D-integrin, FAK and its phosphorylation are not affected in patients with dilated cardiomyopathy (DCM) but are severely reduced in patients with ischaemic cardiomyopathy (ICM), suggesting distinct mechanisms in cardiac remodelling (Pfister et al. 2007). The reduction in β1D-integrin expression in ICM suggests an insufficient adaptation of the failing heart to the ischemic mechanical demand, with the damage being more focal compared with the diffuse disturbance in DCM (Pfister et al. 2007).

Integrin activation is highly regulated by interactions with other cytosolic proteins, particularly talin and kindlins which bind β-integrin tails and can increase integrin affinity for ligands (Calderwood et al. 2013; Rognoni et al. 2016; Zemljic-Harpf et al. 2009). Talin is a bona fide mechanoreceptor, sensing force and converting it into a biochemical response, in a similar way to α-catenin (Han and de Rooij 2016). Both molecules have a C-terminal F-actin binding domain, which can sense force and initiate a conformational change in the molecule, revealing vinculin-binding sites and allowing vinculin recruitment to adhesions (del Rio et al. 2009; Grashoff et al. 2010; Pasapera et al. 2010). During embryogenesis, both talin isoforms, talin1 and talin2, are expressed in cardiomyocytes, but talin2 becomes the main isoform in the adult myocardium, localising at both costameres and intercalated discs (Belkin et al. 1986; Manso et al. 2013; Senetar et al. 2007). Interestingly, cardiac-specific deletion of talin2 in mice results in no significant change in cardiac structure and function, despite a significant reduction in the protein levels of β1D-integrin (Manso et al. 2017). Nevertheless, there is an upregulation of talin1 at the costameres, suggesting that when talin2 is ablated, talin1 can compensate its loss, allowing the heart to maintain its integrity (Manso et al. 2017). An increase in the levels of talin1, but not talin2, was also observed in neonatal rat cardiomyocytes under phenylephrine stimulation, in the hearts of mice after pressure overload and also of human patients with DCM (Manso et al. 2013). These results suggest that while talin2 is the main isoform in the adult myocardium, talin1 can play a role in cardiac mechanotransduction when the heart is under increased mechanical load (Manso et al. 2013; Manso et al. 2017). This role of talin1 appears to be maladaptive since conditional deletion of talin1 in the mouse heart maintained cardiac function and blunted the hypertrophic response after pressure overload (Manso et al. 2013). Specific deletion of both talin isoforms in the mouse myocardium resulted in DCM with a reduction in protein levels of β1D-integrin (Manso et al. 2017). Interestingly, there was a decrease in the vinculin signal at the costameres, but not at the intercalated disc (Manso et al. 2017), supporting the notion that vinculin is anchored to the costameres via talins, but also suggesting that while talin2 is localised at the intercalated disc, vinculin is predominantly recruited there via α-catenin. It will be interesting to see whether talin2 can directly bind vinculin at the intercalated disc and, if so, what are the functional differences compared with the α-catenin-vinculin interaction.

Another protein which can bind the cytoplasmic tail of β-integrin and modulate its activity is the adaptor protein kindlin (Harburger et al. 2009; Rognoni et al. 2016). Kindlin2, the only isoform expressed in the myocardium, was also found to be localised at the intercalated disc and costameres in mouse hearts (Dowling et al. 2008; Hatcher and Basson 2008). Cardiac-specific deletion of kindlin2 in mice results in heart failure, with a significant reduction in the protein levels of β1D-integrin receptors (Zhang et al. 2016). Interestingly, in the hearts of wild-type mice, kindlin2 localisation was found to be exclusive to the costameres, but in kindlin2 knockout mice, there was a decrease in the expression levels of the intercalated disc proteins β-catenin and connexin43 (Zhang et al. 2016). It remains to be established whether kindlin2 is indeed localised at the intercalated disc and, if so, what are its binding partners at this site. One possible candidate is the LIM domain-containing protein migfilin, which was also found to be present at the intercalated disc (Moik et al. 2011) and upregulated in the hearts of mice after pressure overload (Haubner et al. 2015). Similar to talin1, this upregulation appears to be maladaptive, since cardiac function is maintained and hypertrophy is reduced in cardiac-specific migfilin knockout mice after pressure overload (Haubner et al. 2015). Migfilin can bind filamins, thus providing a link between the integrin system and the actin cytoskeleton (Tu et al. 2003). Filamins are large, F-actin cross-linking proteins and also act as scaffolds for a variety of signalling and adaptor proteins, including migfilin (reviewed in Razinia et al. 2012). Since filamins can sense altered mechanical forces and undergo a conformational change, they are believed to act as mechanosensory structures themselves, converting a mechanical signal into a biochemical response (Ehrlicher et al. 2011; Furuike et al. 2001; Razinia et al. 2012). In the adult rat heart, filamin C (filC), a muscle-specific isoform, was found at sites that have to sustain increased mechanical stress including intercalated discs, Z-discs and the sarcolemma (van der Ven et al. 2000). Mutations in filC (reviewed in Verdonschot et al. 2020) were found in patients with hypertrophic cardiomyopathy (Valdes-Mas et al. 2014), restrictive cardiomyopathy (Tucker et al. 2017), DCM (Begay et al. 2016) or forms of DCM with severe arrhythmias (Begay et al. 2018; Hall et al. 2020), suggesting that filC plays a major role in maintaining the structural integrity of cardiomyocytes and ensuring efficient force generation and transmission in the heart muscle. Strong evidence for this hypothesis was provided by a very recent publication, which showed that an inducible cardiac-specific knockout of filC leads to death within a couple of weeks with the hearts displaying a classical DCM phenotype (Zhou et al. 2020).

Melusin, a striated muscle-specific chaperone protein, was also found to bind to the cytosolic domain of β1D-integrin (Brancaccio et al. 2003). Melusin was suggested to act as a mechanosensor, showing an early upregulated expression in the hearts of mice after pressure overload (De Acetis et al. 2005). Mice with cardiac-specific overexpression of melusin maintained adaptive concentric hypertrophy and contractile function in response to pressure overload, preventing left ventricular dilation and the onset of heart failure (De Acetis et al. 2005). By contrast, melusin-null mice fail to retain the concentric compensatory hypertrophic response to pressure overload, rapidly developing left ventricular dilation that evolved into dilated cardiomyopathy (Brancaccio et al. 2003). These results suggest that melusin plays a protective role in the early stages of heart failure (Sorge and Brancaccio 2016). Whether melusin is important for intercalated disc mechanosensing and transduction remains to be elucidated since the protein is mostly found at the costameres (Brancaccio et al. 2003).

Overall, it appears that several components of the integrin-based system (β1D-integrin, FAK, talin2, kindlin2, migfilin, filC) are found at both the intercalated disc and costameres, yet their function in mechanosensing at the intercalated disc remains unclear. It will be important to study these proteins at the cellular level, in confluent cardiomyocytes, which allow cell-cell adhesions to be formed, to carefully dissect their precise localisation, their binding partners and their dynamics at the intercalated disc in response to mechanical force.

### Mechanically gated channels

The large family of transient receptor potential (TRP) channels responds to numerous physical and chemical stimuli via a conformational change to allow cation influx into the cell (Falcon et al. 2019). The TRP vanilloid 2 (TRPV2) channel is a stretch-sensitive, weakly Ca^2+^-selective channel found at the intercalated disc in the murine heart (Iwata et al. 2003). Cardiac-specific deletion of TRPV2 resulted in disrupted intercalated discs, an increased signal of adherens junction proteins (N-cadherin, β-catenin) and cardiac dysfunction (Katanosaka et al. 2014). TRPV2 deletion did not affect excitation-contraction coupling (ECC) which is generated at dyads between the T tubules and the junctional sarcoplasmic reticulum, suggesting that TRPV2-mediated signalling and ECC are spatially and temporally distinct (Katanosaka et al. 2014). A model was proposed where TRPV2 senses force generated from contraction and triggers a biochemical response essential for maintaining intercalated disc structure and thus force transmission between neighbouring cardiomyocytes (Katanosaka et al. 2014). In the hearts of animal models of dilated cardiomyopathy and of human patients with the disease, the levels of TRPV2 are upregulated but relocate to the peripheral membrane (Iwata et al. 2013). The accumulation of TRPV2 at the peripheral sarcolemma was associated with abnormal calcium influx, while the reduced TRPV2 levels at the intercalated disc might be responsible for the changes in cell-cell contact morphology observed in the disease (Iwata et al. 2013; Katanosaka et al. 2014).

## Signalling at the ID

**Fig. 3:**
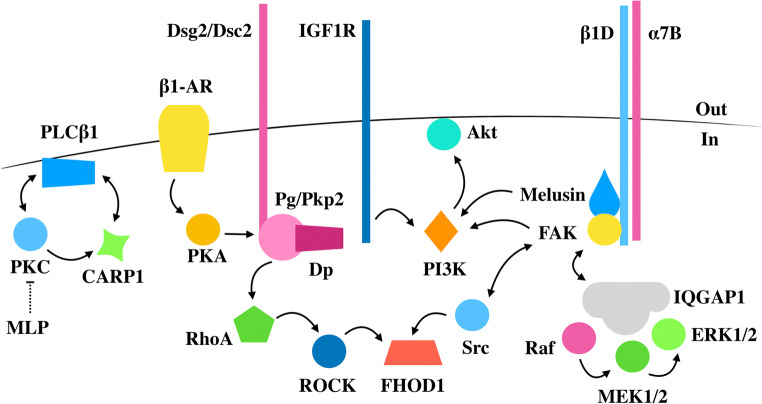
Simplified schematic representation of the signalling molecules modulating the response to mechanical force at the intercalated disc. In the early stages of heart disease, these signalling pathways promote adaptive cell growth and contribute to stronger cell-cell contacts. Aberrant signalling can result in weaker contacts (e.g. in the hearts of TRPV2 knockout mice) and a failure to trigger adaptive hypertrophy (for instance, in the hearts of mice lacking either FAK, melusin or IQGAP1), leading to impaired contractile function. In some cases, impaired cell-cell contacts can result in a complete change in cardiomyocyte identity

The intercalated disc represents a localised subcellular compartment for several signalling pathways which mediate molecular and cellular responses to changes in mechanical force (Fig. 3). The β1-adrenergic receptor is localised at the intercalated disc in the mouse heart (Schlipp et al. 2014). In HL1 cardiomyocytes exposed to β-adrenergic stimulation, protein kinase A (PKA) was found to phosphorylate Pg on Ser665, resulting in recruitment of other desmosomal proteins to cell-cell contacts to increase the intercellular cohesion between neighbouring cells (Schinner et al. 2017). An increase in Pg phosphorylation on Ser665 was also detected in mouse hearts after elevation of cyclic AMP signalling (Yeruva et al. 2020). This mechanism may be an adaptive response to support the increased mechanical load induced by increased adrenergic signalling, which is known to play a role in heart failure (El-Armouche and Eschenhagen 2009). Another signalling component present at the intercalated disc in the failing heart is protein kinase Cα (PKCα) Lange et al. 2016). In a healthy heart, PKCα is inhibited by MLP, but in the hearts of MLP knockout mouse (Ehler et al. 2001) and of human patients with dilated cardiomyopathy where MLP expression is reduced (Zolk et al. 2000), the inhibition is released and PKCα translocates to the intercalated disc, becoming part of a multiprotein complex consisting of cardiac-specific ankyrin repeat protein1 (CARP1), CARP2 and phospholipase C β1 (PLCβ1) (Lange et al. 2016). As elevated PKC signalling plays a critical role in heart failure (Palaniyandi et al. 2009), this provides a molecular mechanism through which the absence or reduced expression of MLP can result in dilated cardiomyopathy.

FAK, bound to the cytosolic tail of β1D-integrin (Pham et al. 2000), acts as a scaffold to recruit several proteins to integrin complexes, including the non-receptor tyrosine kinase Src (Schaller et al. 1994), the p85 catalytic subunit of phosphoinositide 3-kinase (PI3K) (Bachelot et al. 1996) and the adaptor Grb2 (Schlaepfer et al. 1994) (Fig. 3). In the rat myocardium, increased mechanical stress imposed by pressure overload resulted in a rapid phosphorylation of FAK on Tyr397 and assembly of the FAK signalling complex, consisting of Src, PI3K and Grb2 (Franchini et al. 2000). Elevated mechanical demand also activated Akt, an effector of PI3K signalling, and the mitogen activated protein kinase Erk1/2, downstream of Grb2 (Franchini et al. 2000). Since both Akt and Erk1/2 are mediators of hypertrophy (Proud 2004), FAK-mediated signal transduction provides a possible link between increased mechanical load and cardiomyocyte growth (Franchini et al. 2000; Torsoni et al. 2003a). Cardiac-specific deletion of FAK in the adult mouse blunted Erk1/2 phosphorylation levels and the compensatory hypertrophic response to pressure overload, resulting in decreased cardiac function (DiMichele et al. 2006).

In murine hearts, FAK was found to be part of a multiprotein signalling complex consisting of melusin, Raf, MEK1/2, Erk1/2 and the scaffold protein IQ motif containing GTPase activating protein 1 (IQGAP1) (Sbroggio et al. 2011a). Mice with cardiac-specific deletion of IQGAP1 failed to induce adaptive hypertrophy and survival signals in response to pressure overload, accelerating left ventricular dilatation and cardiac dysfunction (Sbroggio et al. 2011b). Since IQGAP1 was also found bound to Akt (Sbroggio et al. 2011b) and melusin to the p85 catalytic subunit of PI3Kα (Waardenberg et al. 2011), the FAK-melusin-IQGAP1 complex appears to play an important role in bringing together and regulating the Akt and Erk signalling pathways in response to increased mechanical demand (Franchini et al. 2000; Sorge and Brancaccio 2016). In mouse L fibroblasts expressing E-cadherins (EL cells), IQGAP1 was found at cell-cell contacts bound to E-cadherin and β-catenin, causing the dissociation of α-catenin from the cadherin-catenin complex and a weakening of cell-cell adhesion (Kuroda et al. 1998). Whether a similar mechanism is present at the intercalated disc remains to be established.

Cardiomyocytes have the ability of secreting the autocrine/paracrine insulin-like growth factor 1 (IGF1) which binds to the IGF1 receptor (IGF1R) to activate the PI3Kα pathway (McMullen 2008; Ren et al. 1999). Neonatal rat cardiomyocytes exposed to IGF1 became enlarged and showed an upregulation of muscle-specific gene transcripts (Ito et al. 1993). Mechanical load imposed by pressure overload resulted in increased IGF1 expression levels in the rat ventricular myocardium (Donohue et al. 1994), and elevated levels of IGF1 were also found in hypertrophied human left ventricles (Pauliks et al. 1999). IGF1 secretion was significantly reduced in cardiomyocytes from TRPV2-deficient mice with impaired cell-cell contacts, in parallel with a significant reduction in IGF1R/PI3K/Akt signalling (Katanosaka et al. 2014). Addition of IGF1 to those cardiomyocytes restored the connection between neighbouring cells, suggesting that the IGF signalling pathway might be involved not only in growth but also in maintaining intercalated disc structure (Katanosaka et al. 2014).

RhoA and its associated kinase (ROCK) signalling are essential in actomyosin machinery assembly, with ROCK phosphorylating and activating myosin regulatory chains but also formins which ensure unbranched F-actin nucleation, elongation and/or bundling (Arnold et al. 2017). In the rat heart, RhoA and ROCK were rapidly elevated in response to pressure overload, clustering to specific subcellular compartments, including the intercalated disc (Torsoni et al. 2003b). RhoA/ROCK signalling appears to be important in FAK-mediated Erk1/2 activation in response to increased mechanical load since RhoA or ROCK inhibition abolished FAK phosphorylation on Tyr397 and Erk1/2 activation (Torsoni et al. 2005). In endothelial cells, ROCK was found to phosphorylate and activate the formin FHOD1, resulting in increased stress fibre formation (Takeya et al. 2008). In cardiomyocytes, FHOD1 was found at the intercalated disc and costameres (Al Haj et al. 2015; Dwyer et al. 2014) with expression levels being elevated in the hearts of mouse models of dilated cardiomyopathy and patients with the disease (Dwyer et al. 2014). Whether the elevated levels of FHOD1 at the intercalated disc are responsible for the increased levels of F-actin observed at this specialised site in dilated cardiomyopathy (Ehler et al. 2001) remains to be established. The non-receptor tyrosine kinase Src, downstream of PKC, was also found to phosphorylate FHOD1, prior to ROCK phosphorylation, suggesting at least a two-step mechanism in FHOD1 activation (Iskratsch et al. 2013; Pandey et al. 2018). Src phosphorylation was shown to be important for formin targeting to costameres (Pandey et al. 2018), but whether a similar mechanism is involved in FHOD1 localisation at the intercalated disc remains to be investigated.

RhoA/ROCK signalling at the intercalated disc appears to play a role in AC (Vimalanathan et al. 2018). A frameshift mutation in the desmosomal protein Pkp2 impaired RhoA/ROCK signalling, triggering transcriptional and morphological changes which govern myocyte to adipocyte transition (Dorn et al. 2018). In human-induced pluripotent stem cells (hiPSC)–derived cardiomyocytes expressing the mutated Pkp2, RhoA recruitment to cell-cell contacts was reduced, resulting in increased levels of cytosolic G-actin (Dorn et al. 2018). In turn, the increased G-actin levels were correlated with cytoplasmic sequestration of transcription factors such as MRTF that are involved in myocyte identity, preventing their entry into nucleus (Dorn et al. 2018; Olson and Nordheim 2010). In the presence of pro-adipogenic cocktails, cardiomyocytes with defective cell-cell contacts failed to sustain myocyte identity and were poised to convert to adipocytes (Dorn et al. 2018), providing a possible molecular mechanism behind the cellular and tissue changes observed in arrhythmogenic cardiomyopathy.

## Conclusion

The intercalated disc contains a complex machinery of structural and signalling molecules which work together to sense, transmit and transduce changes in force to ensure the heart maintains its function in response to increased mechanical stress. It is a fine-tuned machinery and maintained stoichiometry of its composition appears to be crucial for its integrity and heart function. Work carried out in recent years has shown that mechanosensors established by investigating cell-cell contacts in epithelial cells also play a role in mechanotransduction at the intercalated disc, but much still remains to be unveiled. Several questions, such as the precise role of integrin complexes in mechanosensing at the intercalated disc, how desmosomes sense various mechanical loads and whether there are other actin regulatory factors affecting actin structures at this specialised site, will hopefully be answered in the next decade. A clearer understanding of intercalated disc mechanosensing and signalling will allow the developing of pharmaceutical agents that help the failing heart to cope better with the increased mechanical load experienced in disease.
